# Concurrent Squamous Cell Carcinoma and Chronic Lymphocytic Leukemia Presenting as an Enlarging Neck Mass

**DOI:** 10.1177/2324709619842904

**Published:** 2019-04-22

**Authors:** Prabhjot Bhinder, Michael Chahin, Lara Zuberi

**Affiliations:** 1 University of Florida Jacksonville, FL, USA

**Keywords:** chronic lymphocytic leukemia, squamous cell carcinoma, concurrent, oropharyngeal

## Abstract

Chronic lymphocytic leukemia (CLL) patients are at an increased risk for developing more aggressive lymphomas via Richter’s transformation and of developing secondary malignancies. Despite the known association for secondary cancers, oropharyngeal cancers occur rarely. We present a case of a woman with a history of CLL who presented to our facility via transfer for impending airway compromise. Her initial workup was consistent with CLL; however, biopsies were taken of the neck mass because of its aggressive nature. She was treated with rituximab with good response. Final pathology showed evidence of CLL and tonsillar squamous cell carcinoma (SCC). Direct laryngoscopy and further biopsies yielded a diagnosis of unresectable oropharyngeal SCC. She was to be treated with chemotherapy and radiation for her SCC while holding treatment for CLL. This case demonstrates a rare and unexpected concurrent diagnosis.

## Introduction

Chronic lymphocytic leukemia (CLL) is a disorder in which there is an accumulation of mature, monoclonal B lymphocytes. It is the most common leukemia found in Western adults.^1^ According to the National Institutes of Health, CLL comprises 1.2% of new cancer diagnoses in the United States. There is a predominance of males of Caucasian descent, with a median age of 70 years. The clinical course varies with a 5-year survival of 84.2% and median survival of 10 years.^2^ Deaths in patients with CLL are more commonly associated with complications of the disease, such as infection, than from the leukemia itself. Richter’s transformation is an infrequent but highly concerning entity as median survival is about 5 to 8 months.^3^

Regarding complications, CLL has been associated with the development of secondary cancers. In a retrospective study, patients with CLL were shown to have more than twice the risk of development of a second malignancy. This includes solid organs and hematologic malignancies.^4^ Another retrospective study showed an increased risk of solid organs and Hodgkin lymphoma, but not for hematologic cancers.^5^ The cause of this apparent propensity for developing subsequent cancers is unclear. Some suggested theories included genetic predisposition, immunosuppression, and consequences of treatment. However, it is unclear as to whether there is an association with these factors due to conflicting findings.^4-7^

This is a case of a woman with history of CLL and an enlarging neck mass who presented a diagnostic dilemma.

## Case Report

Our patient is a 58-year-old African American woman who initially presented to an outside facility of difficulty swallowing, neck pain, fatigue, and shortness of breath for 3 months. She had bilateral neck swelling, greater on the left side, that started about a year prior to her presentation. This patient also had a 4-day history of productive cough and nasal drainage. She had a 30-pack year history and consumed approximately 12 beers daily for years.

She reported a history of CLL and having been treated with chemotherapy, but she was unsure of the regimen. Examination findings and computed tomography (CT) scan of neck at the outside facility were concerning for impending airway compromise, and she was transferred to our hospital to be evaluated by an otorhinolaryngology or oral and maxillofacial surgery service.

During her evaluation in our emergency department, she was noted to have a muffled voice with mild gurgling of secretions with speech. Large, fixed cervical and submandibular lymph node, and a large left neck mass were present. Enlarged axillary lymph nodes were palpated bilaterally. The uvula and posterior oropharynx were not visible due to her tongue and left neck mass. White blood cell (WBC) count was 83 600/mm^3^. She was started on dexamethasone to reduce the compression of the neck mass on the airway. The oral and maxillofacial surgery service team evaluated her and determined that a definitive airway was not indicated. She was admitted to the medical intensive care unit for close airway monitoring.

**Figure 1.**
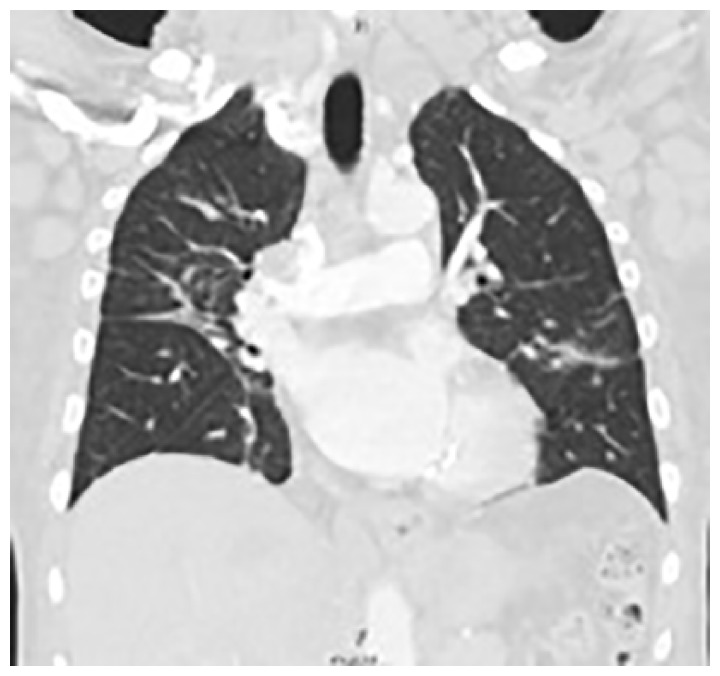
CT Chest with contrast showed consolidative and ground glass opacities and lymphadenopathy.

**Figure 2.**
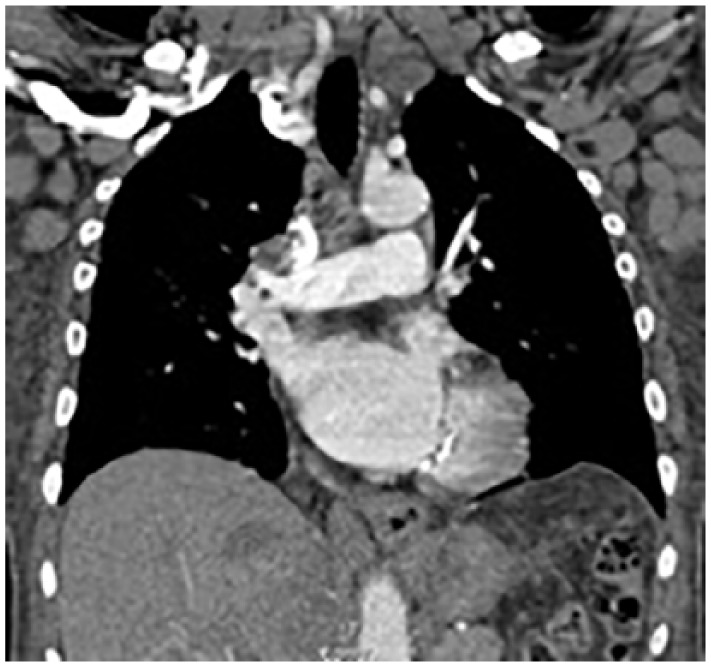
CT Chest with contrast showed lymphadenopathy of mediastinum and right hilum.

The oncology team became involved at this time. Her CLL was the suspected etiology of the neck mass and lymphadenopathy, and intravenous fluids and allopurinol were started to prevent tumor lysis syndrome. Further laboratory workup showed the predominantly lymphocytic, elevated WBC count as before and a normocytic anemia, hemoglobin 10.6 g/dL. Smudge cells were present on the peripheral blood smear. Iron studies, B_12_, and folate were normal. Flow cytometry was pending (Figures 1 and 2).

**Figure 3.**
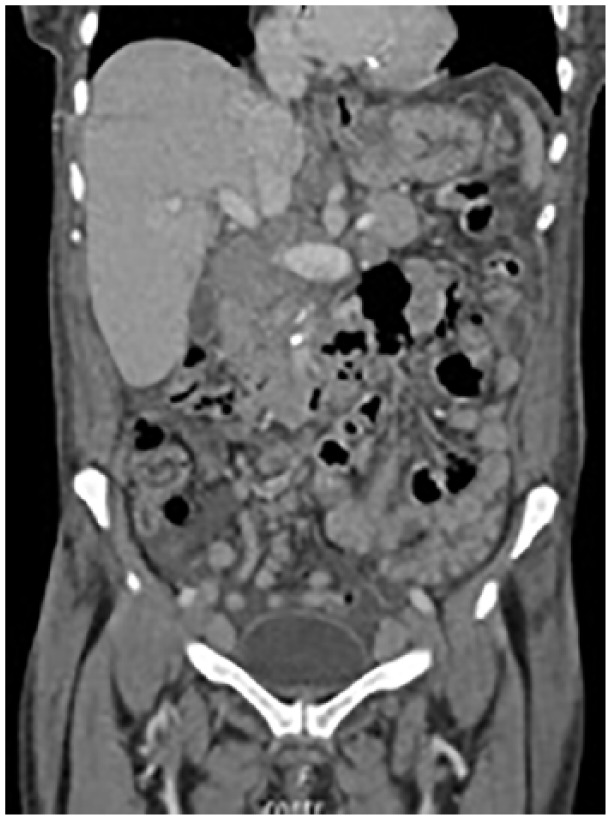
CT abdomen and pelvis with contrast showed confluent retroperitoneal, mesenteric, pelvic, and inguinal lymphadenopathy.

CT chest showed lymphadenopathy of the mediastinum, right hilum, bilateral axilla, submental, and supraclavicular lymph nodes. Consolidative and ground glass opacities were also present in the bilateral lung fields (Figure 3).

Partially confluent retroperitoneal, mesenteric, pelvic, and inguinal lymphadenopathy were noted on CT abdomen and pelvis. These findings were consistent with known CLL.

In addition to CLL, diagnoses considered included Richter transformation. We proceeded to obtain biopsies of the left neck mass.

Around this time outside records were received. Our patient had been treated with rituximab and chlorambucil 5 months prior after presenting with similar symptoms. She had been incidentally found with a WBC count of 40 000/mm^3^ several years before. She was lost to follow-up after both occasions. We started her on rituximab for treating CLL with possible Richter transformation. The tentative plan was to treat with fludarabine, cyclophosphamide, rituximab (FCR) if CLL or rituximab, cyclophosphamide, doxorubicin, vincristine, prednisone (R-CHOP) if Richter transformation had occurred.

Flow cytometry showed a CD5+ mature B-cell lymphoma consistent with CLL, 13q deletion, and mild CD38 positivity. Over the next few days, she had improvement in her neck pain. The mass and lymph nodes decreased in size. The WBC count decreased to 40 000/mm^3^. Biopsy of the neck mass showed squamous cell carcinoma (SCC) with extensive necrosis involving lymphoid tissue.

**Figure 4.**
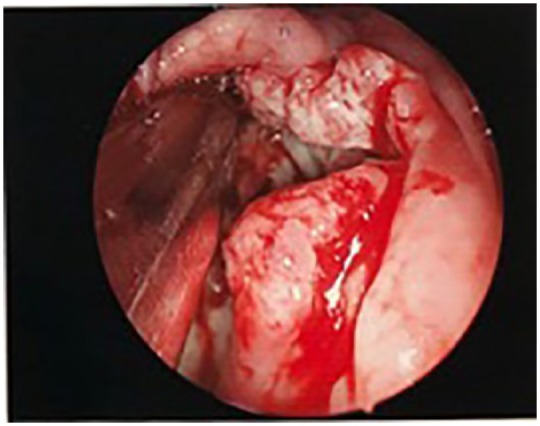
Direct Larynoscpy showed large mass of oral cavity extending to tonsils.

**Figure 5.**
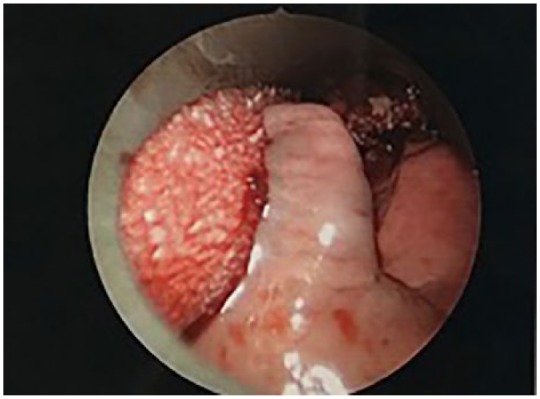
Direct larynxscopy showed mass involving right tonsil.

With evidence of a second malignancy with possible significant oropharyngeal involvement, direct laryngoscopy with biopsy was pursued (Figures 4 and 5).

Direct laryngoscopy was performed and showed a large mass involving the right tonsil extending to midline of the posterior oropharynx. Biopsies were obtained. Posterior oropharynx, right tonsil, and right nasopharynx biopsy contained invasive keratinizing SCC, moderately differentiated, with basaloid features. The nasopharyngeal biopsy also included atypical lymphoid infiltrate consistent with involvement by CLL.

This patient’s case was discussed at the tumor board. Given the widespread involvement of her disease, she was diagnosed with Stage IV oropharyngeal SCC. She was not at candidate for resection and was to be treated with high-dose cisplatin (25 mg/m^2^) weekly for 6 cycles and radiotherapy. Treatment was to be delayed for her Rai Stage III CLL out of priority since her airway compromise was attributed to the SCC and the CLL component had been partially treated with rituximab.

At time of discharge, she had continued improvement of her symptoms. The plan was for her to follow with our medical oncology and radiation oncology teams as an outpatient. However, due to long distance from home, she established care at an outside facility.

## Discussion

After determining whether our patient was able to protect her airway, it was crucial to evaluate for the exact etiology of her enlarging neck mass. One percent to 10% of CLL patients develop Richter’s syndrome, or transformation into an aggressive non-Hodgkin lymphoma.^3^ Though her treatment was palliative in nature, she may have had a worse outcome if only her CLL was considered. There is a case in the literature involving a 62-year-old woman whose airway became compromised by a lymphoid mass related to CLL.^8^

Though there is an association between CLL and the development of second cancers, oropharyngeal SCC rarely occurs. An analysis of 46 164 patients with a histologically confirmed diagnosis of CLL drawn from SEER 18 registries between 2000 and 2014 does not mention oropharyngeal cancer. The 2823 individuals who developed solid tumors were diagnosed with either lung and bronchus, prostate, melanoma, bladder, kidney, or thyroid cancer.^6^ A study that evaluated secondary malignancies in 514 patients with CLL showed 1 case of tongue cancer.^5^ Curiously, another retrospective analysis using SEER data of different regions and timeframes, the longest being 1973 to 2008, showed rare occurrence of oropharyngeal malignancy.^9^

It is difficult to determine what shared risk factors were involved in her developing both malignancies. There is a paucity of similar cases in the literature, and the exact temporality of her diagnoses is unknown due to lack of continuous care. Our patient had a history of tobacco and alcohol abuse, which are known risk factors for oropharyngeal cancer.^10^ Developing CLL is associated with tobacco use.^11^ Though there is a case in the literature of a 52-year-old nonsmoker male who was diagnosed with small lymphocytic lymphoma during workup for an human papillomavirus–positive SCC of tonsillar primary.^12^ Alcohol consumption has not reliably been shown to have positive or negative association with CLL.^13,14^

## Conclusion

In a patient with a rapidly enlarging mass, such as in our case, it is important to rule out causes beyond CLL. It would be prudent to obtain biopsy in cases of uncertain diagnosis given the possibility for Richter’s transformation or a second malignancy.

The development of concurrent oropharyngeal SCC and CLL is exceptionally rare. Given the association of CLL and secondary malignancies, one should be vigilant about continuing to screen patients after diagnosis with CLL. Though the exact cause of this process is not known, it is imperative to continue mitigating possible risk factors such as tobacco smoke exposure. There need to be further studies addressing risk factors associated with CLL and the development of second malignancies so that a better approach may be taken regarding risk reduction.
